# The mouse metabolic phenotyping center (MMPC) *live* consortium: an NIH resource for in vivo characterization of mouse models of diabetes and obesity

**DOI:** 10.1007/s00335-024-10067-y

**Published:** 2024-08-27

**Authors:** Maren Laughlin, Richard McIndoe, Sean H. Adams, Renee Araiza, Julio E. Ayala, Lucy Kennedy, Louise Lanoue, Louise Lantier, James Macy, Eann Malabanan, Owen P. McGuinness, Rachel Perry, Daniel Port, Nathan Qi, Carol F. Elias, Gerald I. Shulman, David H. Wasserman, K. C. Kent Lloyd

**Affiliations:** 1 National Institute of Diabetes and Digestive and Kidney Diseases, Bethesda, USA; 2 Center for Biotechnology and Genomic Medicine, Augusta University, Augusta, USA; 3 Department of Surgery, School of Medicine, University of California Davis, Davis, USA; 4 Center for Alimentary and Metabolic Science, School of Medicine, University of California Davis, Davis, USA; 5 Mouse Biology Program, University of California Davis, Davis, USA; 6 Vanderbilt University, Nashville, USA; 7 Unit for Laboratory Animal Medicine, University of Michigan, Ann Arbor, USA; 8 Department of Comparative Medicine, Yale School of Medicine, New Haven, USA; 9 Department of Internal Medicine, Yale School of Medicine, New Haven, USA; 10 Department of Cellular & Molecular Physiology, Yale School of Medicine, New Haven, USA; 11 Department of Molecular & Integrated Physiology, University of Michigan, Ann Arbor, USA; 12 Caswell Diabetes Institute, University of Michigan Medical School, Ann Arbor, USA

**Keywords:** Diabetes, Obesity, Metabolism, Mouse, Phenotyping, In vivo, Resource, Service, Tests

## Abstract

The Mouse Metabolic Phenotyping Center (MMPC)*Live* Program was established in 2023 by the National Institute for Diabetes, Digestive and Kidney Diseases (NIDDK) at the National Institutes of Health (NIH) to advance biomedical research by providing the scientific community with standardized, high quality phenotyping services for mouse models of diabetes and obesity. Emerging as the next iteration of the MMPC Program which served the biomedical research community for 20 years (2001–2021), MMPC*Live* is designed as an outwardly-facing consortium of service cores that collaborate to provide reduced-cost consultation and metabolic, physiologic, and behavioral phenotyping tests on live mice for U.S. biomedical researchers. Four MMPC*Live* Centers located at universities around the country perform complex and often unique procedures *in vivo* on a fee for service basis, typically on mice shipped from the client or directly from a repository or vendor. Current areas of expertise include energy balance and body composition, insulin action and secretion, whole body carbohydrate and lipid metabolism, cardiovascular and renal function, food intake and behavior, microbiome and xenometabolism, and metabolic pathway kinetics. Additionally, an opportunity arose to reduce barriers to access and expand the diversity of the biomedical research workforce by establishing the VIBRANT Program. Directed at researchers historically underrepresented in the biomedical sciences, VIBRANT-eligible investigators have access to testing services, travel and career development awards, expert advice and experimental design consultation, and short internships to learn test technologies. Data derived from experiments run by the Centers belongs to the researchers submitting mice for testing which can be made publicly available and accessible from the MMPC*Live* database following publication. In addition to services, MMPC*Live* staff provide expertise and advice to researchers, develop and refine test protocols, engage in outreach activities, publish scientific and technical papers, and conduct educational workshops and training sessions to aid researchers in unraveling the heterogeneity of diabetes and obesity.

## Introduction

Understanding the heterogeneous mechanisms behind diabetes and obesity relies heavily on the study and analysis of mouse models that faithfully recapitulate the molecular pathways underlying the pathophysiology of disease in humans. However, the research community faces growing challenges to access, apply, and implement specialized tools and procedures to measure subtle quantitative and qualitative phenotypes in the burgeoning number of genetically-altered (e.g., transgenic, knockout), surgically-manipulated (e.g., bariatric surgery), and environmentally- challenged (e.g., high fat diet) mouse models. Indeed, the expertise, capabilities, and instrumentation needed to conduct complex physiologic, metabolic, and behavioral measurements in live mice might not be readily available at all research institutions engaged in diabetes and obesity research.

In response to these challenges, the National Institute for Diabetes, Digestive and Kidney Diseases (NIDDK) at the National Institutes of Health (NIH) recently launched a new effort to provide centralized mouse phenotyping resources to investigators with the inception of the Mouse Metabolic Phenotyping Centers (MMPC)*Live* Program (RRID:SCR_008997). In 2025 the NIDDK celebrates 75 years of stewardship and service to the people of the United States through its support and conduct of research to improve health and quality of life for people with diabetes and other endocrine and metabolic disorders; liver, intestinal and other digestive diseases; obesity; nutritional disorders; and kidney, urologic, and hematologic diseases, as described in its current Strategic Plan for Research (www.niddk.nih.gov/about-niddk/strategic-plans-reports/niddk-strategic-plan-for-research). NIDDK instituted the MMPC Program in 2001 (Laughlin et al. 2012), and in 2023 it was reimagined as the MMPC*Live* Program to focus on the core strengths and successes of the original program and on the current and future needs of its client user base. MMPC*Live* is intended as a resource to help researchers perform innovative preclinical studies to advance our understanding of biological pathways and environmental contributors to health and disease, particularly those investigators who otherwise would have limited access to the state-of-the-art metabolic and physiologic phenotyping demanded by high quality publications and for successful NIH grant applications. NIDDK intends that MMPC*Live* support the NIDDK research enterprise by providing rigorous and reproducible tests to all mouse researchers, by distribution of high quality technology through standard procedures and training, and by strengthening the research investigator pipeline through enhancing and diversifying the workforce.

**Table:** Box 1. Mouse Metabolic Phenotyping Centers-*Live* at a Glance (www.mmpc.org)

Test catalog	
Application for services	www.mmpc.org/secure/order.aspx
Database	www.mmpc.org/shared/search.aspx
Courses	www.mmpc.org/shared/courses.aspx
Augusta University	www.mmpc.org/
University of California Davis	mmpc.ucdavis.edu/
University of Michigan	mmpc.med.umich.edu/
Vanderbilt University	vmmpc.org/
Yale University	medicine.yale.edu/internal-medicine/drc/resources/institutional/mmpc/
National Institute of Diabetes and Digestive and Kidney Diseases	www.niddk.nih.gov

**Fig. 1:**
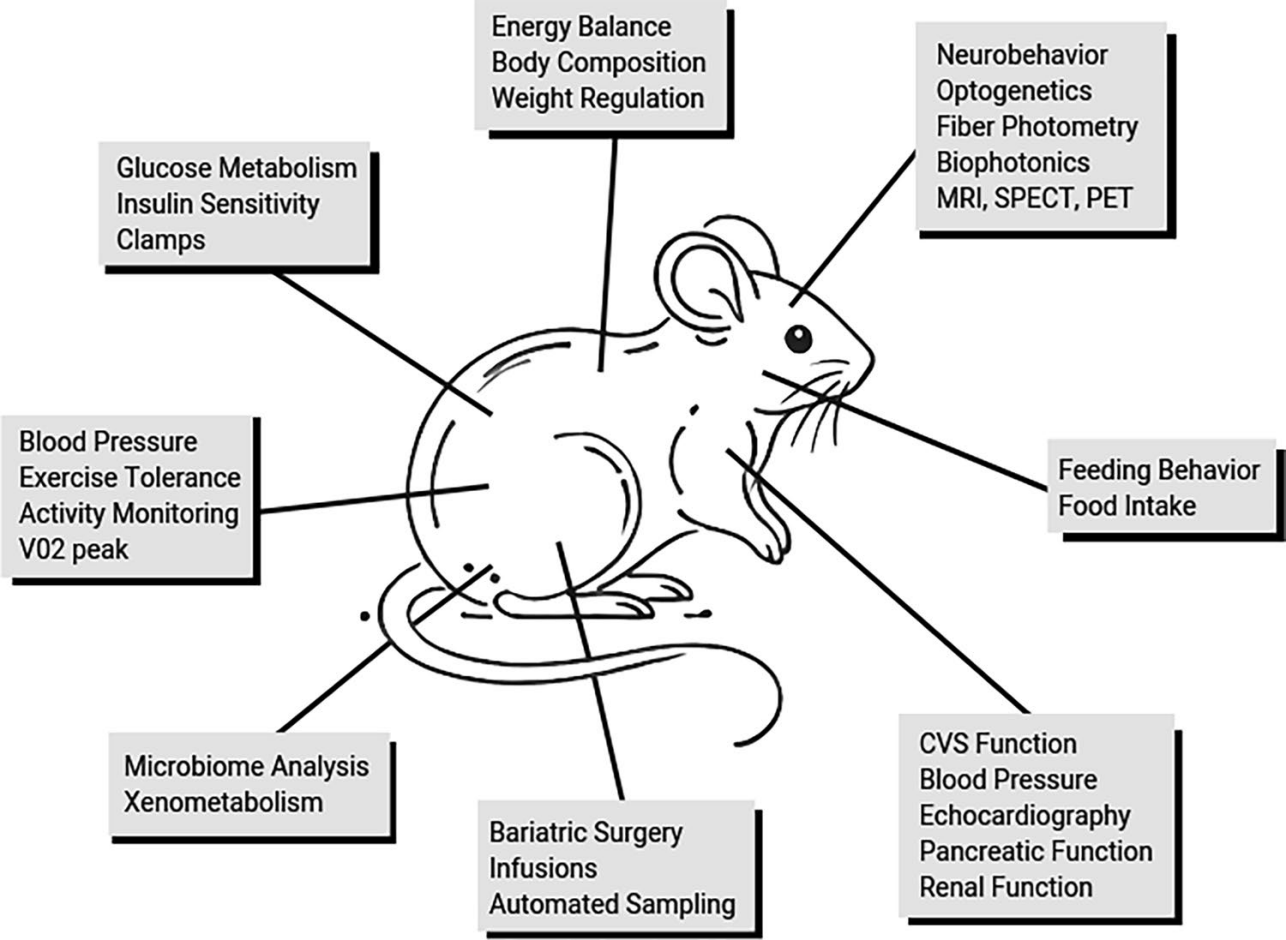
Schematic representation of the variety of tests and procedures available at MMPC*Live* Centers to study the heterogeneity of diabetes and obesity in mice

Emerging after an extraordinarily successful 20 year MMPC Program which offered testing on both mice and other samples (e.g., tissues, plasma), the new MMPC*Live* Program is focused solely on studies conducted on live mice as models of diabetes, obesity, and their complications. Importantly, MMPC*Live* is designed as an outward-facing consortium of collaborating Centers with service cores that provide reduced-cost consultation and standardized metabolic, physiologic and behavioral phenotyping tests on live mice to US academic biomedical researchers. Standardization provides significant benefits to researchers, especially those who either cannot afford specialized equipment or are inexperienced with physiologic testing in live mice. By implementing standardized procedures, MMPC*Live* ensures consistent and reliable data across different studies, enhancing rigor and reproducibility and promoting scientific collaboration. Additionally, standardization reduces costs to MMPC*Live* users by minimizing the need for investigators to obtain expensive, unique equipment and training, thereby making unique, specialized, and complex research capabilities using live mice more accessible to a broader range of institutions (Fig. 1).

## Mission and goals

The mission of the MMPC*Live* Program is to “advance medical and biological research by providing the scientific community with standardized, high quality metabolic and physiologic phenotyping services for mouse models of diabetes, diabetic complications, obesity and related disorders.” To fulfill this mission, MMPC*Live* seeks to achieve four goals:

1. Be responsive to the needs of US academic research community studying the heterogeneity of diabetes and obesity by providing unique and specialized metabolic, physiologic, and behavioral phenotyping on live mice, expert advice and consultation on the use of mouse models, and assistance with experimental design, outcomes measures, and data analysis and interpretation for users, including ARRIVE 2.0 (Percie du Sert et al. 2020), FAIR (Wilkinson et al. 2016), PREPARE (Smith et al. 2018), and LAG-R (Teboul et al. 2024). MMPC*Live* scientists and staff commit to serving all users inside and outside the host institutions equally.
2. Work toward health equity and diversity in the US biomedical research enterprise by supporting historically underrepresented investigators to succeed in biomedical research funding in the areas of diabetes and obesity. MMPC*Live* has established the VIBRANT Program to make resources such as test services, expert advice on experimental design, short internships to learn test technologies available at a heavily subsidized cost as well as to provide enrichment such as travel funding for relevant meetings and expert grant review prior to submission.
3. Support rigor and reproducibility in research by developing and sharing validated protocols for phenotyping live mice, and by providing difficult experimental tests conducted by experts with a high degree of standardization and quality control. By sharing standard operating procedures (SOPs), technologies and web-based tools for gold-standard approaches in experimental methods and data analysis, MMPC*Live* actively contributes to the training of the next generation of research physiologists and metabolic experts.
4. Develop standardized data formats, analytical methods, and storage guidelines for complex data sets, including metadata, for sharing with clients and the public. MMPC*Live* also has adopted the use of unique Research Resource identifiers (RRID; https://scicrunch.org/resources) (Bandrowski and Martone 2016), Digital Object Identifiers (DOI, www.doi.org/), and other best practices directed by the NIDDK Information Network (dkNET, https://dknet.org/) (Whetzel et al. 2015).

## Infrastructure and organization

The MMPC*Live* Program consists of a Consortium of four Centers and a Coordinating Unit (CU) with guidance provided by a Steering Committee of participating principal investigators and NIH officials. The CU provides logistical and administrative support to the Consortium, distributes funds to the Centers, oversees financial management of the VIBRANT program, maintains a dedicated website (www.mmpc.org) with a searchable catalog of tests and procedures, database of phenotyping test results, online data analysis (e.g., energy expenditure), educational, data-sharing, and business tools, and promotes the consortium using a variety of outreach instruments (e.g., exhibit booth and scientific meetings, social media, brochures).

As per instructions in the funding opportunity announcement (RFA-DK-21-027), all Centers in the MMPC*Live* Consortium have a similar organizational structure with a scientific director, technical manager, core leaders, and staff scientists and technicians. Centers are assembled from a cadre of scientific and technical experts, animal and testing infrastructure, and unique and specialized instrumentation and resources to cover the scope of needs across the broad scientific community. Although Centers cooperate to minimize overlap among their service offerings, certain fundamental procedures and areas of expertise (e.g., energy balance) are available at each Center to cover projected demands. Every Center consists of an administrative core that establishes center priorities, handles billing, and manages data and the operations of experimental phenotyping cores, plus an animal core that receives, pathogen tests, and houses mice, and one or more phenotyping cores that provide testing services to mouse researchers in the United States and abroad.

## Best practices

The four Centers that comprise the MMPC*Live* Consortium are the foundation upon which the MMPC*Live* Program is built. These Centers exist at the University of California Davis (RRID:SCR_015357), the University of Michigan (RRID:SCR_015354), Vanderbilt University (RRID:SCR_021939), and Yale School of Medicine (RRID:SCR_006339). The CU is hosted by Augusta University (RRID:SCR_008997). The benefits to the research community derive from the cooperative and complementary nature of a unique national consortium of these regionally-distributed Centers of excellence in diabetes and obesity research using mice. The provision of centralized research resources from a consortium of expert, experienced, and regionally-distributed Centers *broadens the scope, expedites the completion, improves the quality,* and *reduces the overall costs* of scientific studies on live mice to better understand the heterogeneity of diabetes and obesity. MMPC*Live*:

- (1) Enables users access to specialized instrumentation and equipment that are beyond the grasp of individual investigators to enhance the breadth and depth of research infrastructure available for technically complex experiments that would otherwise not be available in individual laboratories without the financial resources, capacity, or expertise to purchase or develop on their own.
- (2) Expedites the likelihood of successfully completing research projects through the participation of a highly experienced and proficient staff and technical experts with longstanding experience conducting unique and complex procedures.
- (3) Facilitates interaction and engagement with staff able to execute specialized studies to make subtle and complex measurements reliably and reproducibly that enhances the accuracy and precision of test results and research outcomes in live mice.
- (4) Relies on highly skilled technical staff to assist with study design, power analysis, experimental planning, test implementation, outcomes measures and interpretation, leading to decreased research costs compared to the time and expense of recreating this specialized scientific environment in one’s own laboratory.

As an NIH-awarded cooperative agreement, programmatic changes made at the Consortium level guide and inform activities at each of the individual Centers. The Consortium is led by a Steering Committee (SC) composed of NIH program officers, external scientific advisors, and representatives of each Center. The SC meets monthly by videoconference and annually at face-to-face meetings to coordinate operations, discuss scientific direction, and address issues and challenges to operations. The SC identifies areas for programmatic development and expansion, including tests and procedures, training workshops, and community engagement. Subcommittees and task forces established by the SC standardize administrative operations and policies on animal husbandry, health, and care, database management, rigor and reproducibility, outreach, and the VIBRANT Program. Local scientific expertise, technical strengths, and capabilities determine the areas of scientific emphasis and technical services available at each MMPC*Live* Center. In this way, one Center might have a unique feature complementary to those at other Centers in the Consortium. An investigator can select from among a broad range of capabilities and expertise across the entire Consortium instead of just one Center to design, plan, and conduct a research study.

## Phenotyping cores

Each MMPC*Live* Center has at least one Phenotyping Core that provides a suite of specialized services and procedures on client’s mice. A phenotyping core is designed to serve investigators from outside the recipient institution with the same priority and at similar cost as those from internal users. Each is headed by a scientific director and technical manager who have the expertise and experience with tests and procedures performed on live mouse models relevant for research in diabetes, obesity, and other complex metabolic diseases. Core personnel can design, adapt, standardize, and validate a variety of tests conducted on mice submitted by clients. Availability of staff and instruments notwithstanding, Centers generally have sufficient laboratory and vivaria infrastructure, testing and service capacity, and expert and experienced staff to manage all incoming requests from users. Centers serve an average of 40–80 users per year. Project scheduling, staggered testing, and cross-training staff on multiple procedures enables Centers to accommodate hundreds of mice at a time dispersed across a variety of testing platforms, such as clamps and infusions, indirect calorimetry, surgical models, glucose tolerance, and telemetry. Relative testing capacities vary between Centers. For example, the MMPC*Live* Center at the University of Michigan can perform indirect calorimetry on ~2240 mice per year on their Sable Promethion system and insulin clamps on ~12 mice per week. On the other hand, the MMPC*Live* Center at Yale can perform indirect calorimetry on ~770 mice per year and insulin clamps on ~32 mice per week.

Phenotyping Cores provide unique and powerful tests that are in demand, relevant, and state of the art, yet not widely available to the investigator community. The range of phenotyping tests available from the Phenotyping Cores across the MMPC*Live* Consortium is extensive. While over 150 tests are offered in the online searchable catalog (www.mmpc.org/shared/orderTestSelection.aspx), a subset of these are requested most frequently (Table 1). These can be clustered into tests of insulin sensitivity and metabolism, pancreatic islet function, body composition and weight regulation, macronutrient oxidation and energy balance, food intake and behavior, cardiovascular function and complications of diabetes, and microbiota characterization including markers of microbial metabolism. However, the long-term success of the MMPC*Live* Program will depend on the extent to which the Centers respond to the needs of the scientific community. Scientists are thus encouraged to inquire if a desired phenotyping test is not listed in the catalog, as Centers will try to accommodate specialized requests.

**Table 1:** Most frequently requested tests and procedures from the 4 MMPC*Live* Centers

University of California, Davis	University of Michigan	Vanderbilt University	Yale University
Body tissue composition and bone mineral density (Echo-MRI/DEXA)	Energy expenditure (indirect calorimetry)	Energy expenditure (indirect calorimetry)	Blood sampling and/or tissue collection
Energy expenditure (indirect calorimetry)	Metabolite turnover and metabolic fluxes (steady-state isotope labeling of metabolites)	Body tissue composition	Body tissue composition
Exploratory locomotor behavior (open field)	Insulin sensitivity (hyperinsulinemic-euglycemic clamp)	Metabolite turnover and metabolic fluxes (steady-state isotope labeling of metabolites)	Metabolite turnover and metabolic fluxes (steady-state isotope labeling of metabolites)
Memory and learning (passive avoidance, Y-maze, novel object recognition, Morris Water Maze)	Automated infusion and blood sampling	Insulin sensitivity (hyperinsulinemic-euglycemic clamp)	Insulin sensitivity (hyperinsulinemic-euglycemic clamp)
Automated infusion and blood sampling	Fat flux (uptake and oxidation) in tissues (^3^H-triolein)	Glucose metabolism (glucose tolerance test, oral/intravenous)	Energy expenditure (indirect calorimetry)
Energy intake and feeding behavior	Bomb calorimetry	Carotid artery blood sampling	Glucose flux in tissues (^14^C-2-deoxyglucose)
Physical performance, exercise, and endurance (running wheel)	Glucose metabolism (glucose tolerance test, oral/intravenous)	Energy Intake and feeding behavior	Glucose metabolism (glucose tolerance test, oral/intravenous)
Glucose metabolism (glucose tolerance test, oral/intravenous)	Cardiovascular health (Treadmill exercise V02 peak)	Cardiovascular health (Treadmill exercise V02 peak)	Fat flux (uptake and oxidation) in tissues (^3^H-triolein)
Bomb calorimetry	Chronic portal vein canulation and sampling	Chronic portal vein canulation and sampling	Physical performance, exercise, and endurance (running wheel)
Cardiovascular health (Treadmill exercise V02 peak)	Memory and learning (Morris Water Maze)	Physical performance, exercise, and endurance (running wheel)	Cardiovascular health (Treadmill exercise V02 peak)

The capacity to make multiple measurements in a single mouse allows for more precise analyses of pathophysiological responses to perturbations leading to diabetes or obesity while also reducing the total number of experimental animals required for a given study. Considering the expense and time involved in shipping mice to a Center for testing, it is cost effective and efficient to obtain as much data from a single mouse as possible. Many tests offered by the Phenotyping Cores are noninvasive or minimally invasive, thus allowing application of multiple assessments on a single mouse. This practice also helps to reduce and refine the number of mice used in research, which is consistent with 3Rs principles (Tannenbaum and Bennett 2015). Some of those tests include tail cuff blood pressure monitoring, echocardiography, urine analysis, exercise tolerance, body composition, food absorption, food intake, fecal energy content, energy expenditure, and activity monitoring. Data obtained from a single mouse are further maximized in terminal procedures by harvesting blood or tissues that can be immediately processed or suitably archived for further analysis. Genetically modified mice can develop unexpected phenotypes that can be detected through a broad range of testing, without compromising the primary research test (e.g., hyperinsulinemic-euglycemic clamp).

An important aspect of the MMPC*Live* mission is the opportunity for researchers to communicate and interact directly with Center scientists and staff with vast experience and novel expertise with mouse studies of diabetes and obesity and on a broad range of phenotyping tests and procedures. The Center can advise investigators on preliminary screening strategies and tests that can be performed and analyzed to inform and guide the plan for secondary and tertiary testing at the Center. For example, an authority on cancer biology might generate a knockout mouse exhibiting hyperglycemia and increased adiposity. The cancer biologist, who would normally think little about the determinants of hyperglycemia and obesity, can initiate an informed investigation on the means by which gene deletion causes the metabolic phenotype by beginning a dialog with one of several MMPC*Live* contacts listed at www.mmpc.org.

MMPC*Live* Centers operate on a fee-for-service basis. Costs for staff, supplies, equipment, and materials are partially underwritten by NIH grants awarded to the Centers which they use to provide limited subsidization of testing and service fees charged to users. Nevertheless, Centers are expected to derive a portion of their operating expenses from user fees charged not only for tests, but also experimental planning advice and consultation on study design, test selection, power analysis, statistics services, outcomes measures, and data analysis and interpretation.

## Animal core

Each Center has an Animal Core that offers processes for clients to ship their mice to the Center for testing. Animal Cores import, house, feed, monitor, and maintain the health of submitted mice for the duration of time required to complete a phenotyping project. Centers will manage importation of mice from investigators depending on their host-institution specific policies and procedures, including veterinary review of animal health records from the user’s institution and inspection of mice upon arrival at the Center’s institution, testing for excluded pathogens, temporary quarantine, etc. Mice sourced from an approved vendor generally avoid quarantine and subsequent testing delays. The Animal Core will provide full animal care, husbandry, and veterinary oversight, including colony management, breeding, cohort expansion, and humane euthanasia. For projects that require testing at more than one Center, users can either ship their mice to one Center which will forward to the second Center, or they can ship their mice to both Centers simultaneously. Generally, mice are not returned at the end of studies, but special arrangements can be made upon request. For example, clients can request mice from an Animal Core with certain surgical procedures, such as bariatric surgery or surgical implantation of intestinal cannula. All research use of mice is first reviewed and approved by the Institutional Animal Care and Use Committee of the Center conducting the testing or service for the client.

Importantly, it is possible for an investigator to use an MMPC*Live* Center even if they do not have a mouse model produced within their own laboratory. For example, several of the nearly 20,000 new mutant mouse models being produced by the International Mouse Phenotyping Consortium (www.impc.org) exhibit phenotypes consistent with diabetes, obesity, diabetic complications, and diabetes-related disorders (Rozman et al. 2018). These mice are available to order and can be submitted to an MMPC*Live* Center for analysis. If mice needed for a study are present at a public repository like the NIH Mutant Mouse Resource and Research Center (MMRRC), then the Center can assist an investigator to arrange for the purchase, shipping, and transportation of mice to a Center for testing. For example, an MMPC*Live* Center and MMRRC are co-located at UC Davis, which makes acquisition of mouse models for analysis seamless. Finally, if mice with the specific characteristics needed for study are not yet available or accessible, then several of the MMPC*Live* Centers have the capability and expertise in genetic modification (e.g., CRISPR genome editing) to produce the mouse models *de novo* for an investigator to use for phenotyping.

Investigators also have the opportunity to work with gnotobiotic mouse models at MMPC*Live* Centers. The ability to evaluate microbiota as a component of a phenotype requires specialized equipment and mice which are available across the MMPC*Live* Consortium. Several common strains of mice are readily available in the germ-free state which can either be used in metabolic studies while remaining germ-free or be reconstituted to a gnotobiotic status with fecal microbiota transplantations. In addition, other transgenic mouse strains of interest can be rederived into a germ-free status and become available for future studies. Additional work has been done to assess the ability to successfully sterilize larger pieces of equipment (e.g., metabolic caging, behavior equipment) to support these studies. Availability of germ-free animals, equipment, and specialized staff remains a barrier for many researchers that are interested in investigating the role of the microbiome in metabolic phenotyping or disease, making this a valuable service to offer to MMPC*Live* clients.

## Administrative core

Finally, each MMPC*Live* Center has its own Administrative Core that is led by the principal investigator and Center director. The Administrative Core manages budgets, staff, workflow, and client interactions, collects fees for services, and manages business and data records. It also oversees collaborations with other Centers. A Local Center Steering Committee made up of the Center director, individual Core leaders, and at least one senior researcher at the host institution who is not part of the Center participate in shared governance and management of the Center, including decision-making regarding test offerings and development, budget and prioritization decisions, and resolving client disputes.

## Data ownership and authorship

MMPC*Live* is designed to provide services at a fair cost to the research community, not to fund the personal or collaborative research programs of MMPC*Live* Center scientists or staff. Investigators can be confident that mice sent to a Center and phenotyping data subsequently derived from testing at the Center belongs to the investigator and does not obligate them to any kind of collaboration, nor are they expected to share authorship with Center personnel when phenotyping data are published. If mutually agreed, collaboration between a user and a Center are allowed as long as NIDDK grant funds are not used in lieu of test fees.

In support of these principles, clients are required to sign a Conditions of Use (COU) Statement (www.mmpc.org/shared/COU.aspx) on the MMPC*Live* website affirming that any data and intellectual property generated by the Center belongs exclusively to the investigator that owns the mouse and his/her institution, not to the Center performing the procedures. Data are returned to the client via the MMPC*Live* database maintained by the CU, using password-protected access. After a reasonable embargo period or publication, test conditions, other metadata, and results can be released and made publicly available online at the request of the client. This process helps investigators fulfill their data sharing and management responsibilities as required by NIH funding support (https://sharing.nih.gov/data-management-and-sharing-policy).

## Vibrant program

MMPC*Live* has established a special program aimed at early career scientists, particularly those from historically underrepresented groups in the biosciences, people from disadvantaged backgrounds, or from institutions that traditionally have not received significant research funding and/or serve historically underrepresented populations. Upon request, VIBRANT-eligible researchers can receive subsidized phenotyping services or can compete for other resources, such as travel and career development awards, expert review of grant applications prior to submission to a funding agency, and consultation and advice regarding experimental design. These resources are expected to improve the ability of these researchers and institutions to compete for research funding in areas relevant to diabetes, obesity, and cardiometabolic health. VIBRANT activities are intended to contribute to building a diverse, high quality national biomedical research force, eventually helping to reduce US health disparities. Each MMPC*Live* Center has developed its own VIBRANT activities relevant to its region, areas of specialty, and institutional relationships. Funding for waived test fees, structured training at the Centers, study consultation, and other forms of career development are funded by special allocations targeted for VIBRANT activities at each Center.

## Specialized technologies

MMPC*Live* Centers each have significant strengths, experience, and capabilities in certain tests and procedures to study mouse models of diabetes, obesity, and other metabolic disorders. These include enhanced *in vivo* imaging resources, glucose-insulin clamps, energy balance, automated blood/body fluids sampling and infusion, optogenetics/chemogenetics, behavioral tests, fiber photometry, and microbiome and indices of microbial metabolism (xenometabolism).

### Enhanced imaging resources

MMPC*Live* enables researchers access to imaging instrumentation and associated infrastructure for metabolic measurements in mice. The breadth of imaging resources and expertise at institutions hosting MMPC*Live* Centers offer unique opportunities for molecular imaging of metabolism at the molecular, cellular, tissue, organ, and whole-body level. Advances in biophotonics, such as laser-trapping Raman and single molecule fluorescence spectroscopy, make possible the nondestructive analyses of individual living cells for characterizing the dynamics of intra-cellular molecular interactions of lipids and proteins. Biophotonics methodologies and magnetic resonance (MR) microscopy offer complementary approaches for tracking cells in real time. MR spectroscopy can be used to obtain real-time measurements of whole body and tissue-specific fat distribution, TCA cycle flux, ATP production, and glycogen synthesis rates. SPECT imaging provides a means to evaluate cardiac function and angiogenesis in relation to diabetes, while PET has been useful for imaging physiological functions including blood flow, glucose metabolism, neurotransmitter synthesis, receptor availability, second messengers, transporters, diffusion, inflammation, and drug delivery. A significant advantage of many of these imaging techniques is that longitudinal studies are possible, and mice can be used later for other phenotyping tests.

### Glucose-insulin clamps

Services that involve clamping plasma glucose and insulin concentrations in awake mice remain among the most heavily used by clients of the MMPC*Live* Centers (Ayala et al. 2010). In the presence of hyperinsulinemia, glucose can be clamped at euglycemia to provide an index of insulin sensitivity, or at hypoglycemia to assess the neuroendocrine response to a fall in blood glucose. Clamping glucose (in the absence of insulin) at hyperglycemia can provide a test of insulin secretion *in vivo*. Considering the utility of these procedures it is of continued interest to devise new ways to improve these approaches. These include combining glucose-insulin clamp technology with isotopes for measuring nutrient flux, mitochondrial metabolism, metabolomics, and microspheres and fluorescence for measuring blood flow.

### Energy balance

Whole-body energy balance is determined by detailed measurements of energy expenditure (EE) and energy intake (EI). The most common approach to estimating EE in mice is to monitor O_2_ consumption rate (V̇O_2_) along with the respiratory exchange ratio (RER; V̇CO_2_:V̇O_2_ ratio), which constitutes indirect calorimetry. There have been rigorous efforts to develop a multi-linear model for calculating EE in mice by incorporating the contributions of both lean and fat compartments (Kaiyala et al. 2010; Kaiyala and Schwartz 2011). MMPC*Live* Centers collaborated with Alexander Banks to develop a web-based calculator (CalR) for performing multi-linear regression to calculate more meaningful values for EE (Rubio et al. 2023) which has now become a standard method for analyzing calorimetry data in mice (https://calrapp.org and https://mmpc.org/shared/regression.aspx).

The other component contributing to energy balance is EI, which is most commonly measured through determination of food intake for individually housed mice. The most sensitive measurement of EI that minimizes technical variance involves multiple measurements of food intake over time, thus enabling calculation of cumulative EI (Ellacott et al. 2010; Ono-Moore et al. 2020). This minimizes reliance on single or few measurements that may not consistently reflect EI. When calculating energy balance, MMPC*Live* Centers also take digestible energy (DE) into account (Ono-Moore et al. 2020), since energy loss via the feces as undigested food energy is critical to understand the net calories available to the body for growth, storage, and maintenance of body functions (notwithstanding difficult-to-measure and smaller energy losses via urine, shedding, and other routes). By quantitatively measuring feces and fecal energy through bomb calorimetry, coupled to EE determinations and careful EI measurements, MMPC*Live* Centers deliver the most state-of-the-art estimations of energy balance in mice.

Although energy balance at the most fundamental level is determined by EE and EI, each of these components are determined by factors such as physical activity, sex, ambient temperature, feeding behavior, strain, and diet. An objective of MMPC*Live* is to further define the effects of these and other variables to help clients understand the mechanisms underlying differences or changes in energy balance in their mouse models.

### Automated blood/body fluids sampling and infusion

MMPC*Live* Centers have Culex/Empis (Basi, Inc.) system stations specifically designed for the automated sampling of serial blood collection or other biological fluids and dosing of pharmaceutical compounds in free-moving unanesthetized rodents (Beier et al. 2007; Gunaratna et al. 2004). The unique design of the Raturn chamber system, which rotates precisely in the opposite direction of the rat or mouse, eliminates the need for fluid swivels. Consequently, a tether to the animal can contain multiple fluid, electronic, or fiber optic lines for the simultaneous sampling of arterial and venous blood, intracerebroventricular fluid, or other bio-fluids. The system allows for no handling of mice during sampling which eliminates much of the stress-induced noise in hormonal measurements and extends sampling capacity from individual animals over a full 24 hours or longer that does not require continuous attention by laboratory personnel. The capabilities of the Culex system for detection of pulsatile hormone secretion in mice without inducing stress was demonstrated by lack of changes in circulating corticosterone levels (Adams et al. 2015; Sáenz de Miera et al. 2024). Applications of this system particularly relevant to MMPC*Live* investigators are serial measurements of circulating ghrelin or other gut peptide levels integrated with feeding events and characterization of pulsatile profiles of counter regulatory hormones including growth hormone, corticosterone, and glucagon. An intragastric dosing catheter can be combined with venous and arterial sampling lines to measure the arteriovenous difference of circulating orexigenic and satiety hormones in response to timed gastric administration of calorically defined meals. Additionally, microdialysis probes can be placed in fat depots or muscle beds to collect samples from the extracellular space to measure the flux of various metabolites in response to feeding or alterations in glucose homeostasis.

### Behavioral tests

MMPC*Live* provides assorted behavioral tests of learning and memory, locomotor activity, anxiety, depression, and more in mouse models of obesity and diabetes. Among these, the most often applied tests are Morris Water Maze (MWM), open field, light/dark box, elevated plus or zero maze, Y-maze, and novel object recognition (Seibenhener and Wooten 2015; Gould et al. 2009; Kelly et al. 2008; Ueno et al. 2020; Miedel et al. 2017; Walf and Frye 2007; Ari et al. 2019; Leger et al. 2013; Lueptow 2017). The Centers have independent video analytical systems (Ethovision XT, Noldus) and CCD cameras for recording, tracking, and analyzing videos of each test. These tests are performed in a dedicated behavioral phenotyping suite with independent sound and lighting control that provides a quiet testing environment and soft, shadow-free ceiling bounce lights with intensity ranging from 20 to 1000 Lux. Tests are individually handled by a designated research assistant during the entire experiment from animal familiarization or acclimation toward the end of the test.

### Fiber photometry and optogenetics

Several MMPC*Live* Centers provide access to custom cellular physiological techniques including *in vivo* intracellular calcium measurements with fiber photometry and *in vivo* optogenetic manipulation of cell activity. These studies take advantage of genetic models developed within individual laboratories in combination with genetically targeted light activated opsins or fluorophores to manipulate and monitor activity from specific neuronal subpopulations of interest. Genetically encoded calcium indicators, such as GCaMP6, are used to measure calcium dynamics, a surrogate of cell activity. The incorporation of these techniques with other assays, such as behavior (e.g., food intake, Y-maze) or metabolism (e.g., glucose clamps or VO2 peak during endurance running), permit real-time investigation of distinct neuronal subpopulations and their role in metabolic processes and whole animal physiology (Flak et al. 2020). In addition to these approaches, the activity of specific neuronal populations can also be modulated using Designer REceptors Activated by Designer Drugs (DREADD) approaches (Bales et al. 2022).

### Microbiome and indices of microbial metabolism (xenometabolism)

Determination of gut bacteria community structures has limited value in linking the microbiome to host health. It is increasingly recognized that microbial metabolism leads to thousands of xenometabolites, a subset of which have signaling and other properties. The MMPC*Live* Center at UC Davis is developing targeted analysis of the bacterial metabolite landscape, also known as xenometabolomics, that can be monitored in mouse models of altered metabolism or diet. Such approaches have previously revealed diabetes-associated xenometabolic signatures in a variety of biospecimens. For instance, progression of diabetes in the UC Davis T2DM rat model led to distinct changes in the cecal metabolome of animals of the same strain, sex, diet, and approximate age (Mercer et al. 2020). Distinct differences in the fecal metabolome across several chemical classes including lipids have also been reported for non-human primates that spontaneously develop T2DM phenotypes (Jiang et al. 2022; Tian et al. 2022) and in feces or stool cultures from humans with T2DM (Zhao et al. 2019; Yang et al. 2022; Xu et al. 2021).

## Browsing, searching, and ordering tests, procedures, and services

The MMPC*Live* CU maintains informatics infrastructure to allow clients to browse, search, and select tests to order from a Catalog of Services (COS) available from each of the Centers. The COS is regularly updated to reflect changes in Centers’ offerings and to accommodate a variety of researchers’ knowledge and background. For example, a user with little experience in metabolic phenotyping might be unsure which tests would be most appropriate to study a particular phenotype while others might have extensive experience and know exactly what to order. The CU has developed three complementary interfaces on its COS: (1) a default search bar for free-text searches; (2) a structured search based on a Center, research areas of interest, test groups, keywords, and/or tissues; and (3) a decision-tree interface in which tests and procedures are suggested to users based on their answers to questions about the phenotype they are interested in testing. The resulting search lists, describes, and shows costs for phenotyping tests and procedures from each of the four MMPC*Live* Centers that match a researcher’s search criteria. Clients can then select and order online their desired phenotyping test(s) from their preferred Center. Once an order is submitted, a notification is sent by email to the appropriate Center whose customer service staff then contact the client to answer any scientific or technical questions, setup a project plan, arrange for shipping of mice, schedule tests, etc. The CU then tracks the order status and links it to testing data and procedural results via the MMPC*Live* website. Centers can use the CU system to attach quotes, invoices, and order receipts.

## Training and education

Through education and outreach efforts, MMPC*Live* Centers serve as a resource to open dialogues on standardization of procedures to study the mouse. For many procedures, the approaches are relatively easy to learn and can be implemented by any laboratory. Others are more complex and require more advanced skills, expertise, and/or equipment. Center scientists and staff have designed and implemented well-known and highly subscribed annual courses. For example, The Vanderbilt MMPC*Live* Center hosts two such courses. The “Isotope Tracers in Metabolic Research: Principles and Practice of Kinetic Analysis” is a week-long course that explores tracer isotope technology and its applications in animals and human subjects, currently in its 21st year. Several webinars are also available online. “Glucose Clamping the Conscious Mouse: A Laboratory Course” is an intensive hands-on course designed for researchers who would like to institute the glucose clamp in their home laboratories. The Vanderbilt MMPC*Live* Center maintains a comprehensive laboratory manual online for *in vivo* testing (http://www.mc.vanderbilt.edu/mmpc) and has a video of the surgery and glucose clamp that can be viewed online (Ayala et al. 2011). Finally, an ‘education portal’ is in development that will consist of a series of webinars on conducting and understanding experiments to measure energy expenditure, and metabolic and glucose homeostasis.

## Rigor and reproducibility

MMPC*Live* Centers have published standard operating procedures for numerous procedures to model and study diabetes and obesity phenotypes in live mice, including measuring food intake (Ellacott et al. 2010) and for performing glucose clamps (Ayala et al. 2010). Individual Center scientists have published papers describing methods (Ayala et al. 2006; Rottman et al. 2007; Shearer et al. 2008; Yin et al. 2011; Kaiyala et al. 2010; Kaiyala and Schwartz 2011; Tong et al. 2010; Jandacek et al. 2004), standards (Ayala et al. 2006; Rottman et al. 2003; McGuinness et al. 2009), and strain comparisons (Qi et al. 2005; Burgess et al. 2005; Berglund et al 2008). In addition, the SC of the MMPCLive has established a Rigor and Reproducibility Working Group of members from each of the Centers who propose, discuss, and implement improvements to SOPs and producing informative webinars on conducting and analyzing energy expenditure experiments.

## Future of the MMPC*Live* program

By engaging scientific and technical experts from across the nation to come together and focus exclusively on providing services, training, and assistance to investigators using live mouse models to study the heterogeneity of diabetes and obesity in humans, MMPC*Live* is providing a highly reputable, unique, and essential resource for the US biomedical research community. An added beneficial feature is the VIBRANT program which is helping expand the diversity of the NIH-funded research workforce to include those from historically underrepresented groups in the biomedical sciences. In these ways, MMPC*Live* will continue to serve the nation’s need for specialized expertise and capabilities in the use of live mouse models to study diabetes and obesity. Looking to the future, MMPC*Live* scientists and staff are pursuing new, innovative approaches that will expand the ability to monitor physiological systems in mice relevant to cardiometabolic health in humans. By coupling well-established and validated approaches with emerging technologies, MMPC*Live* will continue to serve the nation’s need for specialized expertise and capabilities in the use of live mouse models to study diabetes and obesity.

## Data Availability

No datasets were generated or analysed during the current study.
